# Polycythemia Rubra Vera and Sporadic Bilateral Renal Angiomyolipomas: A Case Report

**DOI:** 10.7759/cureus.24030

**Published:** 2022-04-11

**Authors:** James C Barton, Barrett P Cary, Robert M Frederickson

**Affiliations:** 1 Department of Medicine, University of Alabama at Birmingham, Birmingham, USA; 2 Department of Medicine, Southern Iron Disorders Center, Birmingham, USA; 3 Department of Medicine, Brookwood Baptist Medical Center, Birmingham, USA; 4 Department of Radiology, Brookwood Baptist Medical Center, Birmingham, USA

**Keywords:** somatic mutation, polycythemia rubra vera, erythropoietin, erythrocytosis, angiomyolipoma

## Abstract

Polycythemia rubra vera (PRV) is a clonal myeloproliferative neoplasm characterized by autonomous production of erythrocytes, neutrophils, and platelets. Angiomyolipomas (AMLs) are benign renal perivascular epithelioid cell neoplasms of which approximately 80% are sporadic. Here, we report synchronous diagnoses of PRV and asymptomatic sporadic bilateral renal AMLs in a 71-year-old woman. We describe her treatment with phlebotomy and hydroxyurea for PRV and surveillance for renal AMLs. We compare the features and treatment of the present case with those of two previously reported women who also had PRV and sporadic renal AMLs. Finally, we discuss the management and acquired genetic basis of both neoplasms.

## Introduction

Polycythemia rubra vera (PRV) is a clonal myeloproliferative neoplasm characterized by the autonomous production of erythrocytes, neutrophils, and platelets [1]. At the diagnosis of PRV, erythrocytosis, increased neutrophil count, and thrombocytosis are typical, with splenomegaly occurring in two-thirds of patients [1]. In the United States, the standardized prevalence of PRV is 22 per 100,000 [2], and the age- and sex-adjusted incidence rate of PRV during 2001-2012 was 10.9 per million person-years [3]. In 10,812 patients with PRV, the median age at diagnosis was 65 years, and the male-to-female ratio was 1.64:1 [3].

Angiomyolipomas (AMLs) are benign renal perivascular epithelioid cell neoplasms (PEComas) [4]. “Classic” renal AMLs contain dysmorphic blood vessels, smooth muscle cells, and mature adipose tissue [4]. Most subtypes of AMLs have distinctive characteristics on ultrasound, computed tomography (CT), and magnetic resonance (MR) imaging [5]. Approximately 80% of renal AMLs are sporadic, that is, occur in persons without tuberous sclerosis or lymphangioleiomyomatosis [6]. In a case series of sporadic renal AMLs, the mean age at diagnosis was 49 years, there was a predominance of women, and AMLs were bilateral in 13% of patients [7]. In a study of 17,941 ostensibly healthy adults who underwent renal ultrasonography, diffusely hyperechoic masses suggestive of renal AMLs were discovered in 41 (0.23%) study participants of whom 43.9% were women [8].

Here, we report the synchronous diagnoses of PRV and sporadic bilateral renal AMLs in a 71-year-old woman and compare features of the present case with those of two other women aged 79 years and 59 years who also had PRV and sporadic renal AMLs [9,10]. We discuss the management and acquired genetic basis of both neoplasms.

## Case presentation

A 71-year-old non-Hispanic white woman presented with fatigue. Her medical history included transient ischemic attacks, type 2 diabetes mellitus, hypertension, and hyperlipidemia. Her regular medications were aspirin 325 mg, empagliflozin, sitagliptin, amlodipine, hydrochlorothiazide, valsartan, simvastatin, omeprazole, and conjugated estrogens daily and semaglutide weekly. She had western European ancestry. Her sister had Waldenström macroglobulinemia. She had no family history of myeloproliferative neoplasm, tuberous sclerosis, or lymphangioleiomyomatosis. A physical examination revealed no significant abnormality. Laboratory values at diagnosis are displayed in Table 1.

**Table 1:** Laboratory values of a 71-year-old woman with polycythemia rubra vera. Non-fasting serum glucose was 7.21 mmol/L. Other chemistry profile values were within respective reference limits.

Analyte	Result	Reference range
Erythrocytes, ×10^12^/L	6.43	4.20–6.30
Hemoglobin, g/L	149	120–180
Hematocrit, %	47.0	37.0–51.0
Mean corpuscular volume, fL	73.1	80.0–97.0
Red blood cell distribution width, %	18.0	11.5–14.5
Leukocytes, ×10^9^/L	10.0	4.1–10.9
Neutrophils, ×10^9^/L	6.5	2.0–7.8
Platelets, ×10^9^/L	617	140–440
Serum iron, µmol/L	11.6	4.8–24.9
Transferrin saturation, %	14	15–55
Serum ferritin, µg/L	12	15–150
Serum erythropoietin, IU/L	27.4	2.6–18.5

Allele-specific analysis of blood leukocyte DNA detected Janus kinase 2 (*JAK2*) p.V617F (chromosome 9p24, exon 14, c.1849G>T). Ultrasonography revealed normal spleen size and bilateral multiple small hyperechoic lesions of both kidneys. These findings were interpreted as PRV, iron depletion, and sporadic bilateral renal AMLs. She was treated with phlebotomy of 300 mL and hydroxyurea of 500 mg daily. Thereafter, hematocrit remained <45.0%, leukocyte and neutrophil counts remained normal, and thrombocytosis resolved. She did not have another transient ischemic attack.

**Figure 1:**
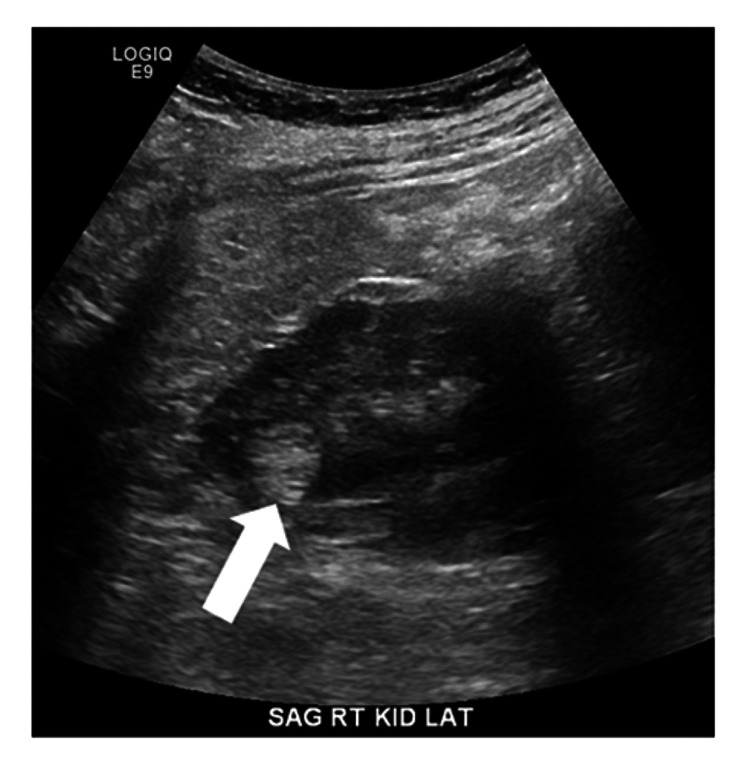
Ultrasound image of the right kidney of a woman with polycythemia rubra vera reveals an echogenic lesion interpreted as angiomyolipoma (arrow).

**Figure 2:**
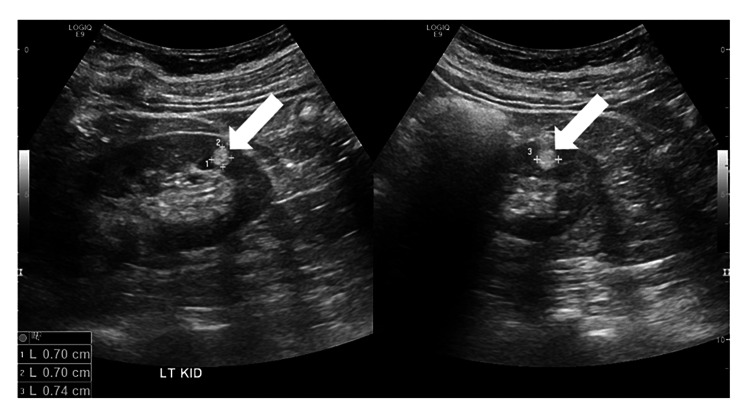
Ultrasound images of the left kidney of a woman with polycythemia rubra vera reveal echogenic lesions interpreted as angiomyolipomas (arrows).

During the 18 months after diagnosis, three additional renal ultrasound studies of variable technical quality revealed bilateral multiple small hyperechoic renal lesions without obvious changes (Figures 1, 2).

**Figure 3:**
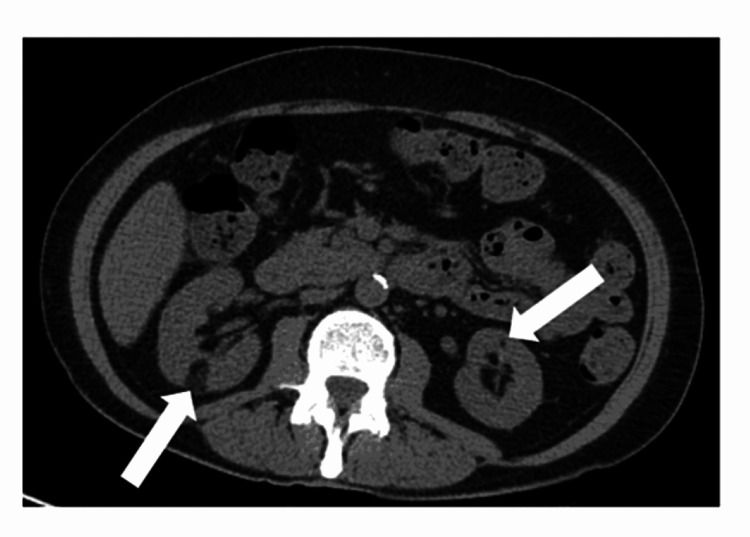
Non-contrast CT image of a woman with polycythemia rubra vera reveals bilateral fat-containing renal neoplasms interpreted as sporadic angiomyolipomas (arrows). The diameter of the largest neoplasm was 1.6 cm (right kidney).

Non-contrast CT imaging of her abdomen and pelvis performed 22 months after diagnosis confirmed normal spleen size and demonstrated two fat-containing masses in the right kidney and three in the left kidney (Figure 3).

## Discussion

The present patient had a hemoglobin of 149 g/L and an elevated erythropoietin (EPO) level at diagnosis of PRV. Hemoglobin levels at diagnosis of PRV in men and women are usually >165 g/L and >160 g/L, respectively [11]. PRV phenotypes are milder in women than in men [12]. Presentation of PRV without erythrocytosis is rare [13]. Subnormal EPO is a minor diagnostic criterion for PRV [1]. EPO levels do not provide additional diagnostic utility for PRV in patients with *JAK2 *p.V617F [14]. Western blot analyses have revealed lower EPO and EPO receptor levels in sporadic renal AML tissue from a woman with PRV than in normal kidney tissue [9].

Spleen size at diagnosis of PRV is usually measured using ultrasound or CT [1]. Thus, ultrasonography in the present case revealed normal spleen size and the unexpected incidental finding of bilateral renal AMLs. In another woman with PRV who had abdominal discomfort and a large abdominal mass, CT imaging revealed normal spleen size and a large renal AML [10].

Management goals for many patients with PRV, including the present patient, incorporate maintaining hematocrit at <45.0% with therapeutic phlebotomy and controlling leukocytosis and thrombocytosis with daily hydroxyurea [1]. The present patient continues to take aspirin daily, consistent with recommendations for patients with PRV in whom thrombocytosis has been controlled and risks for cardiovascular events are increased [1]. PRV in another woman with sporadic renal AML was treated with phlebotomy and hydroxyurea [9]. PRV in a second woman with sporadic renal AML was treated with hydroxyurea alone [10].

The present patient had sporadic bilateral small asymptomatic renal AMLs for which surveillance alone is the preferred management [6,15]. The detection of estrogen receptor-beta, androgen receptor, progesterone receptor, estrogen receptor-alpha, and aromatase in 100%, 79%, 38%, 28%, and 10% of sporadic renal AMLs, respectively, suggests the potential roles of hormones in the pathogenesis and treatment of these neoplasms [16]. It has been postulated that hormones stimulate the proliferation of perivascular smooth muscle and contribute to the pathogenesis of renal AMLs [16]. In a 15-year-old woman with tuberous sclerosis, the rapid growth of a previously stable renal AML that occurred during 12 months of oral estrogen/progestin contraceptive therapy required management with selective arterial embolization [17]. Nonetheless, we found no evidence-based rationale to discontinue hormone replacement therapy for the management of sporadic bilateral renal AMLs in the present case. Two other women with PRV had large sporadic unilateral renal AMLs that were treated with partial or total nephrectomy [9,10].

Radiological classification of sporadic renal AMLs depends largely on imaging features attributable to fat cells [5]. “Classic” AMLs, the most common subtype, have abundant fat and thus marked hyperechogenicity on ultrasound evaluation, similar to those in the present case and in two other women who also had PRV [9,10]. CT or MR imaging is indicated for further evaluation of patients who have either a mass, pain, bleeding, or renal dysfunction possibly due to renal AMLs [7,10] or atypical renal lesions interpreted as “non-classic” AMLs on ultrasound imaging [5,6,8].

*JAK2 *p.V617F was detected in the present patient and two other women who also had PRV and sporadic renal AMLs [9,10]. This somatic mutation occurs in hematopoietic cells of >90% of persons with PRV [1]. A germline single-nucleotide polymorphism in *JAK2 *increases the risk for the development of p.V617F [18]. In one study, first-degree relatives of patients with a myeloproliferative neoplasm had a 5.7-fold increase in the risk of developing PRV [19]. The present patient reported European ancestry whereas two other women with PRV and sporadic renal AMLs were Chinese [9] and Thai [10]. The incidence of PRV is approximately 60% greater in white persons of European ancestry than African Americans or persons of Hispanic or Asian/Pacific ancestry [3]. Thus, heritable factors contribute to the pathogenesis of PRV.

Many sporadic renal AMLs contain somatic mutations of the *TSC2 *gene (chromosome 16p13.3) that encodes tuberin, especially c.5135delC (p.A1712fs) or, less frequently, somatic mutations of the *TSC1 *gene (chromosome 9q34.13) that encodes hamartin [20]. Sporadic renal AMLs have very few mutations in other protein-coding regions [20]. Thus, somatic *TSC2*/*TSC1 *mutations account for the development and progression of sporadic renal AMLs [20], although mutation analyses of *TSC2 *and *TSC1 *were not performed in the present case or reported in two previous similar cases [9,10].

## Conclusions

PRV and sporadic renal AMLs sometimes occur in the same patient. Clinical, laboratory, and radiographic abnormalities in such patients reflect combined characteristics of both PRV and sporadic renal AMLs. Somatic mutations in different genes account for PRV and sporadic renal AMLs. Whereas treatment of PRV often includes phlebotomy and hydroxyurea, management of sporadic renal AMLs is often surveillance.
